# Editorial: Regulatory T lymphocytes in cancer immunity

**DOI:** 10.3389/fimmu.2022.1065570

**Published:** 2022-10-24

**Authors:** Dennis Adeegbe, Joseph Barbi, James Wing

**Affiliations:** ^1^ Department of Immunology, H. Lee. Moffitt Cancer Center, Tampa, FL, United States; ^2^ Department of Immunology, Roswell Park Comprehensive Cancer Center, Buffalo, NY, United States; ^3^ Department of Thoracic Surgery, Roswell Park Comprehensive Cancer Center, Buffalo, NY, United States; ^4^ Human Immunology Team, Center for Infectious Disease Education and Research (CiDER), Osaka University, Osaka, Japan

**Keywords:** regulatory T cells, KLRG1, branched chain amino acid (BCAA), hematologic malignances, Foxp3 interactome, black raspberries

In this Research Topic, several authors delved into pivotal aspects of regulatory T cell (Tregs) biology with added emphasis on cancer immunity. While Tregs are critical for maintenance of immunological self-tolerance, they play a suppressive role in anti-tumor immunity (1). The biology of Tregs has been extensively described but their molecular features as it relates to other effector cells continue to unravel. The report by Borys et al. highlights the importance of KLRG1, an inhibitory co-receptor in the biology of Tregs as well as effector T and NK cells. While we have a better understanding of the role of KLRG1 in the latter, there is paucity of knowledge on this issue in the former especially in the context of anti-tumor immunity. Historically, KLRG1 signaling restrains the effector activity of subsets of effector T and NK cells (2, 3) and the existing dogma is that it is associated with highly differentiated cells. In this regard, one may argue that KLRG1 expression may identify effector and regulatory T cells that are “in action” during an active inflammatory response in which case, targeting KLRG1 may be a tug of war between its functional role in effector cells versus regulatory T cells. It is anticipated that ongoing efforts will shed more light on how this co-inhibitory receptor functions in Tregs, information that would be instrumental on the net effect of KLRG1 targeting for immunotherapeutic purposes. With KLRG1 already in clinical development, it would be interesting to see whether its application in investigational therapeutic settings has as much promise as other immunomodulatory agents such as PD-1, CTLA-4, and TIGIT (4, 5) or even newly emerging natural compounds such as black raspberry extract.

The benefits of the phytochemicals and phytonutrients found in dietary fruits and vegetables have been suggested by numerous studies, and their potential roles in preventing chronic conditions such as diabetes, obesity, cardiovascular disease, and potentially cancers are being explored (6). Studies of phytochemical- and antioxidant-rich black raspberries are suggesting anticarcinogenic effects and cancer prevention utility in colorectal, esophageal, skin, and oral cancers (Huang et al.; 7, 8). While both chemoprevention and immune-modulating properties have been seen in clinical and mouse model studies, the cellular and molecular mechanisms involved are not completely defined. As described by Ryan et al., black raspberry extract can potentiate the inhibition of Treg recruitment into head and neck squamous cell carcinoma mouse models. Interestingly, the disruptive effect by this extract was specific to Tregs and did not extend to effector T cells which were demonstrated to display enhanced anti-tumor cytotoxic activity. It will be interesting to see whether this compound could be widely applicable as a tumor-Treg targeting agent in other cancers.

Metabolic wiring of tumor-infiltrating Tregs is an active area of investigation and distinct immunometabolic pathways are operative in these cells presumably as an adaptation to the “not-so’ conducive” tumor microenvironment as summed up by Yahsi and Gunaydin Metabolism of Branched chain amino acids (BCAA) such as isoleucine have demonstrated effects on tumor-infiltrating Tregs and existing studies suggest this intersects with mTORC1 and Wnt signaling axes through intermediate effectors such as TCF-1 to control downstream mitochondrial metabolic processes. Accumulation of Lipids in Tregs through CD36 may oppose the breakdown of BCAA revealing an intricate web of metabolic pathways that control the suppressive function of tumor-associated Tregs. As the field of metabolomics continue to evolve, better understanding of metabolites from BCAA catabolism could reveal the Achilles heel of tumor-Tregs, representing opportunities for tumor-specific Treg-targeting (9–11).

Another succinct yet comprehensive review by Maharaj et al. discusses the role of Tregs with respect to their prognostic value, origin, phenotype and function in B-cell lymphoid malignancies which are a heterologous group of common cancers often associated with poor outcomes. While in many cases Tregs hinder the anti-cancer immune response, their presence may also indicate a more active immune response making their use as a prognostic indicator complex. Indeed, in the case of follicular lymphoma a higher proportion of Tregs are associated with increased survival (12) while in chronic lymphocytic leukemia (CLL) a higher proportion of Tregs is seen in cases with poor outcomes (13–16). This complexity may also be reflective of the complex interplay between the tumor microenvironment and the Tregs themselves. Of particular note to the control of B-cell malignancies is the existence of a subset of Tregs with specialized function for the control of B-cells, the T-follicular regulatory cells (Tfr). These cells were initially described based on expression of the chemokine receptor CXCR5 and their ability to travel to the B-cell follicle where they act to dampen germinal center responses and plasma cell development (17, 18). Tfr can be found in a range of B-cell malignancies and have distinct characteristics and transcriptional signatures to Tregs. While it may be expected that Tfr may have a more specialized role in the control of follicular lymphoma as the authors point out, further research is still required to fully separate their function from that of Tregs due to the high level of phenotypic similarity the two.

In hematologic malignancies, Tregs express high levels of the immunosuppressive molecule CTLA-4 which is key to their suppressive program. However, it is not yet clear if targeted inhibition of Tregs by the anti-CTLA-4 antibody ipilimumab is effective in hematologic malignancies such as B-cell lymphoma and non-Hodgkin’s lymphoma as levels of Tregs appear to fluctuate under this therapy (19). From mouse studies as pointed out by authors, it appears that the makeup of the other cells in the tumor microenvironment is critical to the outcome of anti-CTLA-4 mediated immunotherapy.

For some time, it has been appreciated that the generation of Tregs and their characteristic suppressive function depend on the complex interactions of diverse molecular players. For instance, the DNA binding domain of Foxp3 has been characterized as having a relatively low affinity for DNA, and this master regulator of Treg biology appears to rely on an expansive company of interaction partners to access target genes and regulate their expression (20, 21). Within the growing “Foxp3 Interactome” (22) are transcription factors like NFAT, AML1/Runx1, Eos, and Gata3 (20, 23–25) and enzymes capable of executing post-translational modification of histones and Foxp3 itself (i.e., Histone/protein Deacetylases and Histone/protein Acetyltransferases, E3 ligases, and deubiquitinases). Prior studies have identified several Foxp3-interacting co-factors as key facilitators or enforcers of Treg function and identity (26). Others have even documented how sterically altering the access of certain co-factors to the Foxp3 protein complex can result in context-specific effects on Treg function in vivo that can impact pathologies like diabetes and arthritis in preclinical models (27, 28). It is clear that targeting key protein complexes in Tregs may allow for therapeutic relief of excessive immune suppression in the cancer setting. However, priority targets sensitive to current therapeutic tools need to be identified and characterized in order to pursue this strategy. In their review, Christensen and Hancock describe and discuss six so-called “Coregulatory Complexes” known or suspected to impact the differentiation, function, or stability of Tregs by interacting with Foxp3 or otherwise altering transcriptional gene expression mechanisms. The authors summarize the well-documented roles played by select elements of the epigenome regulating machinery in the regulation of Foxp3 gene expression and Treg function. A number of recent studies are discussed calling attention to the importance of these complexes for Tregs and their constituent factors, including Histone/protein Deacetylases expected to modulate both chromatin accessibility and Foxp3 stability in Tregs. Importantly, the potential value and means of targeting elements of these multi-protein complexes to therapeutically undermine Treg-mediated immune suppression and improve outcomes in cancer patients are also discussed.

As our knowledge of Tregs in cancer immunity continue to evolve, the immediate future is expected to see concerted and focused efforts in the area of Treg-focused approaches leveraging molecular, metabolic, genomic and epigenomic features of tumor-associated Tregs for their selective targeting to create a more permissive anti-tumor effector response in support of therapeutic end goals.

## Author contributions

DA, JB, and JW contributed equally to the writing, editing, and review of the article. All authors contributed to the article and approved the submitted version.

## Acknowledgments

We thank all participating authors for their critical contributions to this Research Topic.

## Conflict of interest

The authors declare that the research was conducted in the absence of any commercial or financial relationships that could be construed as a potential conflict of interest.

## Publisher’s note

All claims expressed in this article are solely those of the authors and do not necessarily represent those of their affiliated organizations, or those of the publisher, the editors and the reviewers. Any product that may be evaluated in this article, or claim that may be made by its manufacturer, is not guaranteed or endorsed by the publisher.
